# Inhaled Pro‐Efferocytic Nanozymes Promote Resolution of Acute Lung Injury

**DOI:** 10.1002/advs.202201696

**Published:** 2022-07-20

**Authors:** Haiying Ji, Chengmi Zhang, Fengying Xu, Qianyun Mao, Ran Xia, Muqiao Chen, Wei Wang, Shunan Lv, Weiwei Li, Xueyin Shi

**Affiliations:** ^1^ Department of Anesthesiology and Intensive Care Unit Xinhua Hospital School of Medicine Shanghai Jiaotong University Shanghai 200092 China; ^2^ Department of Anesthesiology No. 971 Hospital of People's Liberation Army Navy Qingdao 266000 China; ^3^ Department of Endocrinology Xinhua Hospital School of Medicine Shanghai Jiaotong University Shanghai 200092 China

**Keywords:** acute lung injury, antioxidant nanozyme, apoptotic cell membrane, efferocytosis, macrophage polarization

## Abstract

Acute lung injury (ALI) is a significant contributor to the morbidity and mortality of sepsis. Characterized by uncontrolled inflammation and excessive inflammatory cells infiltration in lung, ALI has been exacerbated by impaired efferocytosis (clearance of apoptotic cells by macrophages). Through specific receptor recognition and activation of downstream signaling, efferocytic macrophages promote resolution of inflammation by efficiently engulfing dying cells, avoiding the consequent release of cellular inflammatory contents. Here, inspired by the intrinsic recovery mechanism of efferocytosis, an apoptotic cell membrane (ACM) coated antioxidant nanozyme (AOzyme) is engineered, thus obtaining an inhalable pro‐efferocytic nanozyme (AOzyme@ACM). Notably, AOzyme@ACM can efficiently increase apoptotic cell removal by combing enhanced macrophages recognition of “eat me” signals through apoptotic body mimicking and scavenge of intracellular excessive reactive oxygen species (ROS), a significant barrier for efferocytosis. AOzyme@ACM can significantly inhibit inflammatory response, promote pro‐resolving (M2) phenotype transition of macrophage, and alleviate ALI in endotoxemia mice compared with AOzyme group. By addressing the critical factor in the pathogenesis of sepsis‐related ALI through restoring efferocytosis activity, the ACM‐based antioxidant nanozyme in this study is envisioned to provide a promising strategy to treat this complex and challenging disease.

## Graphical abstract

Sepsis‐related acute lung injury is exacerbated by impaired efferocytosis. Inspired by the intrinsic recovery mechanism of efferocytosis, an inhalable pro‐efferocytic nanoparticle is developed. The apoptotic body mimicking nanoparticle is constructed by antioxidant nanozyme coated with apoptotic cell membrane. Enhanced “eat‐me” signals recognition and ROS scavenging synergistically restore defective efferocytosis capacity and ameliorate inflammation.

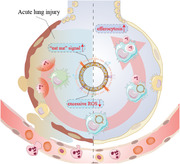

## 1 Introduction

Acute lung injury (ALI) is the process that underlies dysregulated inflammatory immune response and remained the leading cause of death in sepsis.^[^ ^1^ ^]^ Rapid recruitment and activation of neutrophils and proinflammatory (M1) macrophages in the lung is a central process in the pathogenesis of sepsis related ALI.^[^ ^2^ ^]^ LPS‐induced ALI in mice showed direct evidence that if apoptotic neutrophils are not efficiently cleared, they may induce lung proinflammatory cascade owing to secondary necrosis.^[^ ^3^ ^]^ The clearance of apoptotic cells by phagocytes, mainly macrophages, occurs by a process called efferocytosis.^[^ ^4^ ^]^

Efferocytosis acting to ameliorate inflammatory cascade by preventing the secondary necrosis of dying cells and the release of proinflammatory cellular contents, is a key mechanism in the resolution of inflammatory processes.^[^ ^5^ ^]^ Meanwhile, the efferocytic process actively polarizes the macrophage to a pro‐resolving phenotype (M2) which facilitates tissue repair and suppresses inflammation.^[^ ^6^ ^]^ In fact, increasing evidences suggest that causative factors of ALI can lead to impaired efferocytosis.^[^ ^7^ ^]^ Unfortunately, related events within macrophages usually not only downregulate efferocytosis but also increase polarization toward inflammatory phenotype. For example, high mobility group box protein 1 (HMGB1) binding to advanced glycation end products (RAGE) or *α*V integrins blocks macrophage recognition of apoptotic cells, and also reinforces an inflammatory state of macrophage via stimulating NF‐*κ*B.^[^ ^8^ ^]^

Previous observations found that excess reactive oxygen species (ROS) production in macrophage led to increased active Rho and decreased efferocytosis.^[^ ^9^ ^]^ Thus, antioxidant exposure can increase the ability of macrophages to clear apoptotic cells and thereby elicit an anti‐inflammatory effect.^[^ ^9c^ ^]^ To date, the development of enzyme‐mimicking nanomaterials (nanozymes) with good ROS scavenging capacity is a promising approach for the treatment of inflammation.^[^ ^10^ ^]^ Nanozymes have attracted widespread attention due to a variety of advantages, such as broad‐spectrum antioxidative stress properties, robust anti‐inflammatory activity, facile fabrication, and excellent biocompatibility.^[^ ^11^ ^]^

Apoptotic cell membrane (ACM) has multiple ways to provide “eat‐me” signals, such as presenting phosphatidylserine (PS), modifying surface molecules (such as conversion of CD50 to CD31) or changing the components of plasma membrane (such as loss of asymmetry of plasma membrane). These evaluated “eat‐me” signals and downregulated “don't eat‐me” signals (such as suppression of CD47 expression) can attract macrophages and promote their engulfment.^[^ ^12^ ^]^ We have long known that the attachment of “eat‐me” molecules to their receptors on the surface of macrophages, is sufficient to generate intracellular signal cascades and transcriptional programs alterations to enhance efferocytosis and anti‐inflammatory responses within macrophages.^[^ ^13^ ^]^ Given that, some “eat‐me” molecules have been used to modify drug carriers to target phagocytes and promote efferocytosis. For instance, liposomes supplemented with PS, the most prominent and well‐known “eat‐me” signal, are applied in considerable researches.^[^ ^14^ ^]^ However, there are many mechanisms that can inhibit or interfere with PS recognition, such as masking of PS on apoptotic cells by soluble RAGE and annexin A5 or cleaving the PS by neutrophil elastase.^[^ ^8^, ^15^ ^]^ Furthermore, Suggs and colleagues demonstrated that liposomes modified by the combination of membrane‐associated proteins and PS performed better than PS alone.^[^ ^16^ ^]^ Similarly, Nakagawa and colleagues have found NPs that mimicked more precisely the real ACM could result in more effective improvement of efferocytosis.^[^ ^17^ ^]^ These discoveries suggest that real ACM coating may be a better therapeutic intervention due to inheriting natural surface of ACM which contains intact efferocytosis associated ligands.

**Scheme 1:**
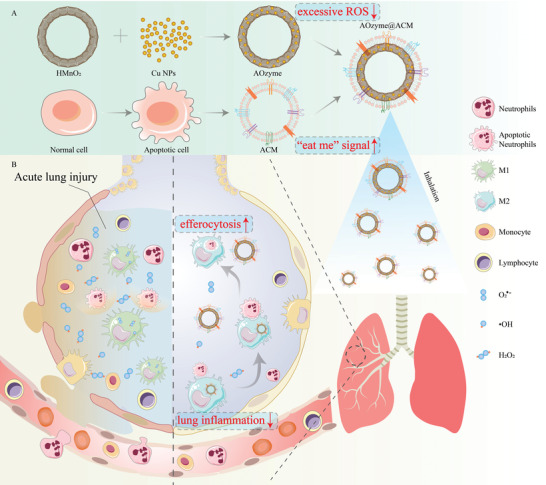
A) The synthesis of apoptotic cell membrane (ACM) coated antioxidant nanozymes (AOzyme@ACM). B) Schematic illustration of the inhaled AOzyme@ACM for resolution of acute lung injury (ALI) through restoring the impaired efferocytosis of macrophage.

So far, most studies on regulation of innate immune cells in ALI have concentrated on mediating the dysregulated migration, recruitment, and inflammatory cytokine release properties of macrophages and neutrophils, while little focused on how to correct the defective efferocytosis. Herein, we for the first time developed a kind of inhalable pro‐efferocytic nanoparticles (NPs). As we know, Cu NPs simultaneously possess glutathione peroxidase‐, superoxide dismutase‐, and catalase‐ mimicking enzyme properties to scavenge hydroxyl radical (•OH), superoxide anion (O_2_^•−^), and hydrogen peroxide (H_2_O_2_).^[^ ^18^ ^]^ Additionally, hollow MnO_2_ NPs (HMnO_2_), which synthesized through gently etching a silica nanoparticles core, not only act as nanocarrier but also have the effect of decomposing H_2_O_2_.^[^ ^19^ ^]^ Therefore, the small Cu NPs (about 2.9 nm) were loaded onto the mesoporous structure of HMnO_2_ to obtain the composite NPs (HMnO_2_—Cu), which modified with polyethylene glycol (PEG) to constitute the biomimetic antioxidant nanozyme (AOzyme) with broad‐spectrum ROS scavenging ability. Eventually, HMnO_2_─Cu was coated by real ACM derived from differentiated PLB‐985 cells (a neutrophil‐like cell line) to form the pro‐efferocytic NPs (AOzyme@ACM). In vitro and in vivo results have showed the AOzyme@ACM inheriting abundant “eat‐me” signals from the source cells could improve the defective efferocytosis of macrophage. Furthermore, the inhalation therapy enabled well‐controlled administration of drug in lung and minimized systemic off‐target effects. This strategy of restoring the “appetite” of macrophages can inhibit inflammatory response, promote pro‐resolving phenotypic shift of macrophage and alleviate ALI in model mice (**Scheme** **1**).

## 2 Results and Discussion

### 2.1 Synthesis and Characterization of Nanoparticles and AOzyme@ACM

In this study, uniform HMnO_2_ NPs were fabricated as seeds via a template‐sacrificing process. First, 100 nm of SiO_2_ NPs were synthesized by Stöber method and positively charged SiO_2_—NH_2_ NPs were obtained by (3‐aminopropyl) triethoxysilane (APTES) modification (Figures S1,S2, Supporting Information).^[^ ^20^ ^]^ The negatively charged MnO_2_ was adsorbed on the surface of SiO_2_—NH_2_ NPs by electrostatic adsorption to form a core–shell structure, which diameter was increased to 130 nm and the Zeta potential was reduced to −30 mV (Figures S1,S2, Supporting Information). HMnO_2_ was obtained by etching SiO_2_ core with sodium carbonate as previously described.^[^ ^19a^ ^]^ As shown in the transmission electron microscopy (TEM) images, the resulting HMnO_2_ had uniform distribution and the thickness of MnO_2_ shell was about 15 nm (Figure S1, Supporting Information). As shown in the X­ray photoelectron spectroscopy (XPS) spectrum, the characteristic peaks at 642.1 and 653.8 eV corresponded to the Mn (IV) 2p3/2 and Mn (IV) 2p1/2 spin‐orbit peaks of MnO_2_, implying the +4 valence state of manganese in the NPs (Figure S3, Supporting Information).^[^ ^19b^ ^]^

**Figure 1:**
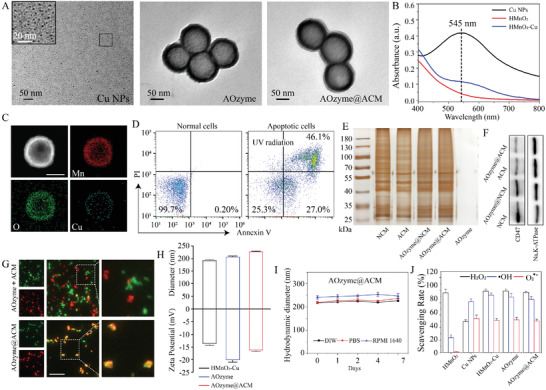
Fabrication and characterization of antioxidant NPs. A) Representative TEM images of Cu NPs, AOzyme, and AOzyme@ACM. B) UV–vis absorption curves of Cu NPs, HMnO_2_, and HMnO_2_—Cu. C) STEM images of the as‐prepared HMnO_2_—Cu, showing the element distribution of Mn, O, and Cu. Scale bar, 50 nm. D) Apoptosis induction of differentiated PLB‐985 cells was analyzed by flow cytometry. E) SDS‐PAGE analysis of retention proteins from NCM, ACM, AOzyme@NCM, AOzyme@ACM, and AOzyme. F) Western blot analysis of the CD47 expression of NCM, AOzyme@NCM, ACM, and AOzyme@NCM, using Na, K‐ATPase as a reference protein. G) Fluorescent microscopy images of either a mixture of AOzyme and ACM (AOzyme +ACM) or of the AOzyme@ACM, the ACM and AOzyme were labeled with Dil (red) and FITC (green), respectively. Scale bar, 20 µm. H) Hydrodynamic diameters and Zeta potential distribution of HMnO_2_‐Cu, AOzyme, and AOzyme@ACM. I) Dispersion stability of AOzyme@ACM stored in water or PBS (pH 7.4) or RPMI 1640 medium. J) H_2_O_2_, •OH, and O_2_^•−^ scavenging capabilities of HMnO_2_, Cu NPs, HMnO_2_‐Cu, AOzyme, and AOzyme@ACM. Data are presented as mean ± SD (*n* = 3).

Next, ultrasmall Cu NPs were synthesized by a green, economic, and simple method. Copper chloride was reduced to Cu NPs by high concentration of ascorbic acid at 80 °C. After the addition of l‐ascorbic acid, the reaction solution gradually changed from colorless to yellow, orange, brown, and finally close to dark color, which is a typical color change process of copper NPs synthesis (Figure S4, Supporting Information).^[^ ^21^ ^]^ UV absorption spectrum showed that Cu NPs had obvious absorption peak at around 545 nm, which is typical UV absorption peak of Cu NPs (**Figure** **1**B).^[^ ^21^ ^]^ TEM images showed that the size of Cu NPs was about 2.9 nm (Figure 1A; Figure S5, Supporting Information).

To obtain HMnO_2_—Cu composite, Cu NPs were adsorbed into the skeleton of HMnO_2_ by electrostatic adsorption. The Zeta potential of the composite decreased from +22 mV of HMnO_2_—NH_2_ to −13.9 mV, which was close to the potential value of pure Cu NPs, indicative of the successful imbedding of Cu NPs (Figure S2, Supporting Information). UV absorption spectrum showed that HMnO_2_—Cu had weak absorption peak at around 545 nm, which is the absorption peak of Cu NPs (Figure 1B). In addition, scanning transmission electron microscopy (STEM) exhibited the uniform distribution of Mn and O elements in HMnO_2_ (Figure S6, Supporting Information). As for HMnO_2_—Cu, the obvious Cu signal in addition to the common Mn and O signals was one of the evidences supporting the formation of HMnO_2_—Cu. (Figure 1C). Furthermore, HMnO_2_—Cu showed two weak signals at binding energies of 932.5 eV and 952.4 eV, which correspond to the binding energies of Cu2p3/2 and Cu2p1/2 in Cu NPs, respectively, indicating that HMnO_2_—Cu was successfully constructed (Figure S7, Supporting Information). ICP‐AES quantitative analysis showed that the mass fraction of copper element in the HMnO_2_—Cu composite nanoparticles was about 4.4%w/w. To improve its colloidal stability and biocompatibility for further application in vivo, PEG molecules were used to modify HMnO_2_—Cu to obtain AOzyme. After PEG modification, the Zeta potential of nanoparticles increased from −35.6 mV to −20.2 mV, indicating that the nanoparticles surface was partially covered by PEG (Figure S8, Supporting Information).^[^ ^20^ ^]^ More importantly, the PEG‐modified nanoparticles remained stable in PBS or cell culture medium, with no significant change in hydrodynamic size during 7 days of storage (Figure S9, Supporting Information). In contrast, the unmodified nanoparticles rapidly agglomerated to 600–800 nm in physiological media and could not meet the requirements for in vivo applications.

Next, we induced apoptosis via UV irradiation in differentiated PLB‐985 cells (about 73% Annexin V positive) (Figure 1D). The ACM was acquired from apoptotic PLB‐985 cells though an extrusion method and was coated onto HMnO_2_—Cu cores to synthesize AOzyme@ACM. To compare and evaluate the advantages of ACM coating, normal cell membrane (NCM) obtained from normal differentiated PLB‐985 cells was used to construct AOzyme@NCM as a control in a similar way. The complete encapsulation through cell membrane could endow HMnO_2_—Cu with superior colloidal stability (Figure 1I). The hydrodynamic diameter of the AOzyme@ACM increased by 21 nm compared with that of the uncoated HMnO_2_—Cu cores; the surface potential was less negative than the cores but comparable to ACM alone (Figure 1H). TEM images further showed they were spherical core‐shell structures, and the surface was uniformly covered by a membrane with a thickness of about 7.6 nm (Figure 1A).

In addition, whole protein analysis of the obtained membranes and the derived NPs were analyzed through sodium dodecyl sulfate‐polyacrylamide gel electrophoresis (SDS‐PAGE). As exhibited in Figure 1E, the silver staining result revealed that the profile of proteins retained on NPs was similar to that of the cell membrane, suggesting the successful membrane coating on NPs. Also, in cells undergoing apoptosis, besides redistribution, CD47, a well‐known “don't‐eat‐me” signal, can also be reduced.^[^ ^22^ ^]^ The western blot analysis showed that CD47 was significantly downregulated in the ACM and AOzyme@ACM bands compared to NCM and AOzyme@NCM, indicating the characteristic protein from cell membranes were inherited by NPs (Figure 1F). Besides, a significant colocalization of fluorescent signals between FITC labeled NPs (green) and Dil labeled ACM (red) further verified the successful membrane coating on NPs. In stark contrast, the mixture of AOzyme and ACM exhibited separated red and green punctate (Figure 1G).

Moreover, it was found that AOzyme@ACM remained stable over 7 days of incubation in PBS and cell culture medium, confirming its great colloidal stability in the physiological environment (Figure 1I).^[^ ^23^ ^]^ The cell counting kit‐8 (CCK‐8) assay against Raw264.7 cells (a murine macrophage cell line) displayed high cell viability, demonstrating the AOzyme@ACM displayed satisfactory biocompatibility, which probably benefiting from the green constituents and assembly process (Figure S10, Supporting Information).

### 2.2 ROS Scavenging Activities and Cytoprotection against ROS Damage of AOzymes

In addition to triggering an excessive inflammatory response, overproduced ROS can inhibit the clearance of apoptotic cells by activating the guanosine triphosphatase (GTPase), Rho, a key negative regulator of phagocytosis.^[^ ^9b^ ^]^

Cu NPs exhibit a wide range of ability to scavenge ROS, including H_2_O_2_, O_2_^•−^, •OH, and other free radicals.^[^ ^18^ ^]^ MnO_2_ was well known for effectively eliminating H_2_O_2_. To test the free radical scavenging ability of the NPs, we evaluated and compared the scavenging behavior of Cu NPs, HMnO_2_ NPs, and HMnO_2_—Cu NPs toward H_2_O_2_, O_2_^•−^, and •OH, respectively (Figure 1J). The efficiency of H_2_O_2_ scavenge was obtained by directly measuring the concentration of hydrogen peroxide before and after the reaction (Figure S12, Supporting Information).^[^ ^18a^ ^]^ For the O_2_^•−^ scavenge experiment, the reaction system of purine and xanthine oxygenase were used to produce O_2_^•−^, and after the experiment was completed, electron transfer substances and color developer were added, and the UV absorbance was measured by spectrophotometer to obtain the O_2_^•−^ content (Figure S11, Supporting Information).^[^ ^24^ ^]^ While methylene blue (MB) can be degraded by •OH but not by H_2_O_2_, it was often used as •OH indicator. Therefore, the change of •OH production can be reflected by detecting the change of MB concentration before and after the reaction (Figures S13,S14, Supporting Information).^[^ ^25^ ^]^

The results showed that Cu NPs could scavenge H_2_O_2_, •OH, and O_2_^•−^ with scavenging rates of 49.5%, 81.4%, and 54.3%, respectively (Figure 1J). HMnO_2_ had a high scavenging efficiency of 95.7% on H_2_O_2_ and a certain scavenging ability on •OH, which might be due to their competition with Fenton's reagent (Fe^2+^) for H_2_O_2_ rather than direct removal of •OH. HMnO_2_—Cu had the advantages of both HMnO_2_ and Cu NPs, and the scavenging rates of the three kinds of free radicals were 98.3%, 91.7%, and 51.7%, respectively. Thus, HMnO_2_—Cu was a highly efficient and broad‐spectrum ROS scavenger. And the ROS scavenging activity of nanoparticles did not change significantly after PEG modification or coating of cell membranes, due to the fact that small molecules of free radicals can freely cross the sparse PEG or phospholipid bilayer (Figure 1J).^[^ ^26^ ^]^ Furthermore, AOzyme can be rapidly degraded in weak acidic solution (pH 6.5) containing 100 µm of H_2_O_2_, which would greatly facilitate the elimination of NPs from the organism after their functions are fully performed (Figure S15, Supporting Information).^[^ ^27^ ^]^

**Figure 2:**
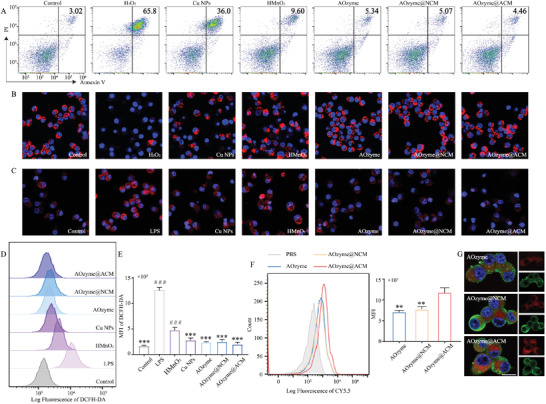
Multienzyme‐like antioxidative activity of AOzyme@ACM in vitro. A) Representative flow cytometry plots assessed by Annexin V and propidium iodide (PI) double‐staining of cell apoptosis distribution of Raw264.7 cells under different treatment condition with exogenous H_2_O_2_ (500 µm) stimulated or not. B) The mitochondrial transmembrane potential (red) of Raw264.7 cells under different treatment condition with exogenous H_2_O_2_ (500 µm) stimulated or not was observed by CLSM. C) Representative images of O_2_^•−^ staining (red) of Raw264.7 cells under different treatment conditions. D) Representative flow cytometry histograms and E) mean fluorescence intensity (MFI) analysis of ROS production by Raw264.7 cells under different treatment condition with LPS (100 ng mL^−1^) stimulated or not. F) Flow diagrams with MFI analysis and G) CLSM images of Raw264.7 (stained with *β*‐actin (green)) incubated with Cy5.5 (red) labeled NPs for 60 min. Scale bar, 10 µm. Data are presented as mean ± SD (*n* = 3). **p < 0.01 and ***p < 0.001 versus LPS. ^###^ *p* < 0.001 versus AOzyme@ACM (one‐way ANOVA with Bonferroni post hoc test).

Previous researches have indicated that both endogenous and exogenous oxidants could inhibit the engulf of apoptotic cells by macrophages.^[^ ^9^ ^]^ We applied Raw264.7 macrophages exposed to oxidants to confirm the cytoprotective properties of NPs against oxidative stress in vitro. First, we examined the cytoprotection against the oxidative damage induced by exogenous ROS (H_2_O_2_ and •OH). Exogenous ROS can induce obvious cytotoxicity because it can easily pass through the cytomembrane. We found AOzyme attenuated H_2_O_2_‐mediated oxidative damage and maintained cell viability more effectively than Cu NPs or HMnO_2_ (Figure S16A, Supporting Information). Highly toxic •OH was generated in the reaction between H_2_O_2_ and Fe^2+^ (Fenton reaction). AOzyme induced an apparent increase of cell viability for •OH‐induced cytotoxicity (Figure S16B, Supporting Information), indicating the stronger capability to eliminate ROS. Furthermore, AOzyme co‐culture reduced the ratios of late apoptotic cells (Annexin V+/PI+ cells) relative to Cu NPs or HMnO_2_ treatment, also confirming the optimized ROS scavenging ability of compound AOzyme (**Figure** **2**A; Figure S17, Supporting Information). Furthermore, the ability of AOzyme@NCM and AOzyme@ACM to reduce ROS cytotoxicity was comparable to that of AOzyme, again confirming that appropriate surface modification does not affect ROS scavenging activity. The destruction of mitochondrial transmembrane potential was known to be one of the earliest events in apoptosis. Confocal laser scanning microscopy (CLSM) analysis revealed that mitochondrial membrane potential vanished less in AOzyme, AOzyme@NCM, and AOzyme@ACM groups than Cu NPs or HMnO_2_ group (Figure 2B), further validating that composite nanozyme could mitigate oxidative stress more effectively.

We further examined the effect of antioxidant NPs on protecting against intracellular endogenous ROS (induced by LPS stimulation). As shown in Figure 2D and 2E, the intracellular ROS level detected by 2′,7′‐dichlorofluorescein diacetate (DCFH‐DA) increased dramatically after LPS treatment. In comparison, the mean fluorescence intensity (MFI) of DCFH‐DA significantly decreased when the cells were pretreated with AOzyme, highlighting augmented ROS scavenging activity of AOzyme. In addition, the ROS level in AOzyme@ACM group was unexpectedly slightly lower than that in AOzyme, which may be due to the activation of the anti‐inflammatory pathway of macrophages by the stimulation of apoptotic cell membranes.^[^ ^28^ ^]^ This trend was further confirmed by the intracellular O_2_^•−^ levels observed via microscopy images (Figure 2C). The above results indicated that composite nanozyme was expected to be efficient in scavenging intracellular ROS which impeding the process of efferocytosis.

At the same time, we wondered if uptake of AOzyme@ACM by macrophage was affected by increased interactions between “eat me” ligands and efferocytic receptors. We then compared the uptake of NPs by Raw264.7 macrophages. Here, Raw264.7 macrophages were analyzed using flow cytometry after incubating with Cy5.5 labeled NPs for 1 h. A higher MFI was observed in AOzymes@ACM group relative to AOzyme (Figure 2F). Interestingly, cell membrane coating alone might not be attributed to this difference, as AOzymes@NCM could not increase macrophage uptake (Figure 2F). Images of CLSM also intuitively exhibited stronger intracellular Cy5.5 fluorescence (red), which was consistent with the results of flow diagrams and MFI (Figure 2G). These results proved the seductive “eat me” signals displayed on ACM effectively facilitated the internalization of NPs by macrophages, thereby improving the targeted intracellular delivery of antioxidant nanozymes.

### 2.3 Pro‐Efferocytic AOzyme@ACM Prevented Inflammation

**Figure 3:**
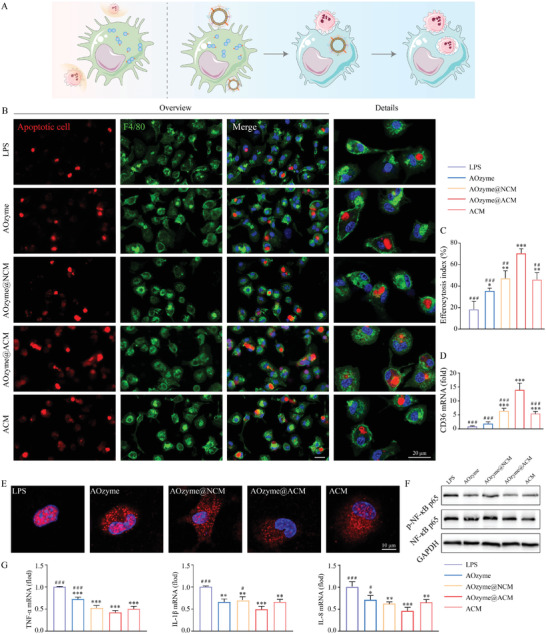
AOzyme@ACM enhanced efferocytosis and alleviated inflammation in vitro. A) Schematic illustration of restoring defective efferocytosis by AOzyme@ACM. B) CLSM analysis to evaluate efferocytosis by PMs in vitro. F4/80 antibody in green; apoptotic Jurkat cells in red; nucleus of PMs in blue. Scale bar, 20 µm. C) Quantitative analysis from three independent experiments was shown in bar graph (Upper right). D) The mRNA expression of CD36 in Raw264.7 cells under different treatment condition upon LPS (100 ng mL^−1^) stimulation. E) CLSM analysis on the nuclear translocation of p65 (red) of Raw264.7 cells stimulated by LPS (100 ng mL^−1^). Scale bar, 10 µm. F) Immunoblots showing phosphorylation of p65 (p‐p65) and p65 expression in Raw264.7 cells challenged with LPS, with GAPDH as a loading control. G) The mRNA expression of TNF‐𝛼, IL‐1*β*, and IL‐8 in Raw264.7 cells under different treatment condition upon LPS (100 ng mL^−1^) stimulation. Data are presented as mean ± SD (*n* = 3). *p < 0.05, **p < 0.01, and ***p < 0.001 versus LPS. ^#^ *p* < 0.05, ^##^ *p* < 0.01, and ^###^ *p* < 0.001 versus AOzyme@ACM (one‐way ANOVA with Bonferroni post hoc test).

As mentioned previously, we speculated that AOzyme@ACM combining pro‐efferocytic ACM coating and multienzyme‐like antioxidative activity could efficiently correct the impeded efferocytosis (**Figure** **3**A). To verify this hypothesis, we isolated peritoneal macrophages (PMs) from mice and exposed them to NPs prior to culturing with LPS. Jurkat T cells (a classical apoptotic cell model for efferocytosis) were labeled with CellTracker CMPTX red dye and then exposed to UV irradiation to induce apoptosis. First, it was observed that PMs exposed to either AOzyme or ACM elicited a slight increase phagocytosis of apoptotic cells, suggesting that ROS scavenging and enhanced “eat me” ligands recognition cooperatively boosted efferocytosis (Figure 3B,C). It was further confirmed that PMs cultured with AOzyme@ACM efferocytosed more apoptotic cells than other NPs, as evidenced by the proportion of F4/80^+^ PMs that engulfing apoptotic cells (Figure 3B,C). Flow cytometry analysis also showed enhanced uptake of CFSE labeled apoptotic cells by AOzyme@ACM treated Raw264.7 cells compared with other treatments (Figure S18, Supporting Information). CD36, a well‐known efferocytic receptor, was more highly expressed in Raw264.7 cells exposed to AOzyme@ACM compared with other NPs, further supporting the pro‐efferocytic function of AOzyme@ACM involving “eat‐me” signal presentation and antioxidant activity (Figure 3D).

Considering the suppressive effects of efferocytosis on inflammation usually depend on the downregulation of proinflammatory signaling, we focused on the role of NF‐*κ*B. In vitro co‐incubation experiments confirmed that the inhibitory effect of AOzyme@ACM on p65 nuclear translocation in Raw264.7 cells exposed to LPS was the most potent among all treatments (Figure 3E). Immunoblot (Western Blot) analyses revealed the protein expression level of p65 phosphorylation (p‐p65), which caused p65 nuclear translocation and transcriptional activity, indicative of NF‐*κ*B pathway activation. Treatment of Raw264.7 macrophages with AOzyme@ACM prior to culturing with LPS resulted in a greater reduction of p‐p65 expression in comparison with other treatments (Figure 3F). Next, the anti‐inflammation effect of NPs was also confirmed in vitro. Apparently, all treatments reduced the production of proinflammatory cytokines (TNF‐a, IL‐1*β*, and IL‐8), and AOzyme@ACM group performed best, which was in line with the negative regulation of NF‐*κ*B activation (Figure 3G). Collectively, our data demonstrate that ACM‐coated antioxidant nanozymes are potent inducers of efferocytosis.

**Figure 4:**
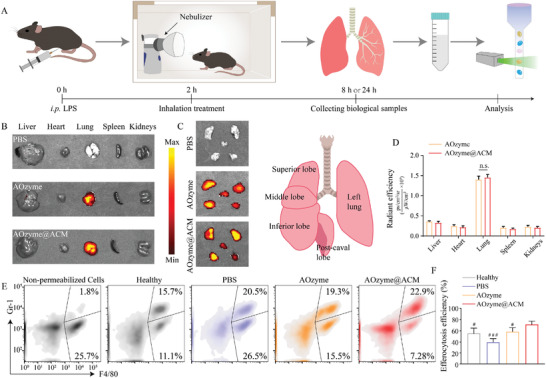
Inhalable AOzyme@ACM reversed impaired efferocytosis in vivo. A) Schematic showing the animal study design. Mice were injected intraperitoneally with LPS (10 mg kg^−1^) 2 h prior to inhalation with NPs or PBS. 24 h later, the BALF was collected and analyzed. B) In vivo fluorescence images of AOzyme and AOzyme@ACM in the indicated organs and C) their biodistribution in different lung lobes 6 h after the inhalation and D) quantitative assessment (*n* = 5). E) Cells collected from BALF were immunostained for F4/80 and intracellular Gr‐1 and subjected to flow cytometric analysis. Left flow cytometry plot (non‐permeabilized cells) showing that macrophage permeabilization is needed to detect Gr‐1, indicating that the Gr‐1 is intracellular, a marker of macrophage engulfment of neutrophils. F) Quantification of the percentage of Gr‐1^+^ cells within F4/80^+^ macrophages (*n* = 5). Data are presented as mean ± SD. n.s. means not significant. ^#^ *p* < 0.05, ^###^ *p* < 0.001 versus AOzyme@ACM (one‐way ANOVA with Bonferroni post hoc test).

Following the in vitro findings, we determined to compare efferocytosis between AOzymes@ACM and AOzyme in vivo. In this experiment, we first investigated whether NPs could successfully reach the inflammatory lungs by inhalation. Cy5.5‐labeled AOzymes@ACM and AOzyme were administered to mice by inhalation for 20 min. 6 h later, the lungs were collected and imaged by an ex vivo imaging system (Figures 4A).

As expected, intense fluorescence intensity was detected in lung and almost absent in other organs (**Figure** **4**B,D). To further estimate the distribution throughout the lung, the lung was dissected into five constituent lobes and homogeneous fluorescence signal was achieved in all lobes of the lung (Figure 4C). In conclusion, the results suggested that NPs could be uniformly dispersed in all lung lobes by inhalation without systemic distribution, indicating excellent availability and potential in noninvasive administration for lung injury. Next, we explored the impact of NPs on efferocytosis in BALF collected from ALI mice. In this assessment, pulmonary macrophages were labeled with anti‐F4/80 antibody; intracellular neutrophils, with anti‐Gr‐1 antibody. We observed a significant increase in macrophages phagocytosis of Gr‐1‐positive neutrophils compared with AOzyme or PBS‐treated mice. (Figure 4E,F). Taken together, the above results suggested that the display of “eat‐me” signals and the elimination of intracellular excessive ROS could upregulate efferocytosis capacity in a coordinated manner.

### 2.4 Pro‐Efferocytic AOzyme@ACM Promoted Macrophage Polarization to M2

There are two distinct phenotypes of activated macrophage, including pro‐inflammatory M1 type and reparative M2 type. Promoting an M1‐to M2‐phenotype shift could benefit inflammatory homeostasis in the lung. We hypothesized that the effect of AOzyme@ACM on efferocytosis could promote macrophage polarization toward an M2 phenotype.

**Figure 5:**
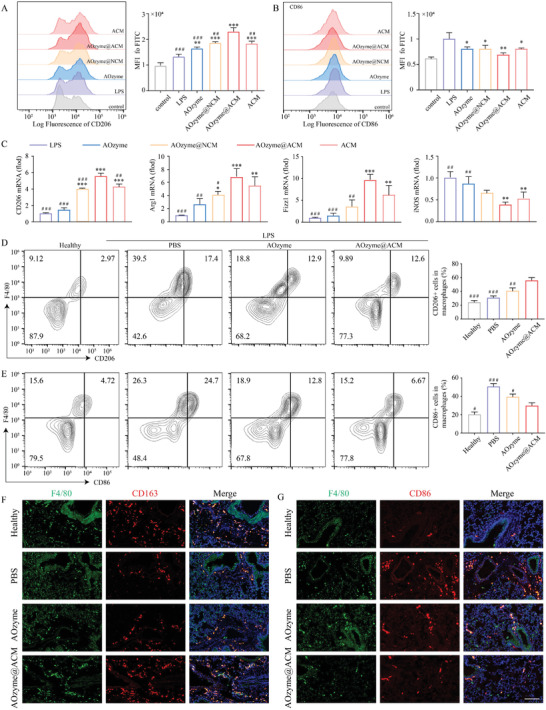
Pro‐efferocytic AOzyme@ACM promoted macrophage M2 phenotype transition. Representative flow cytometry histogram and quantitative evaluation of A) CD206 (an M2 marker) and B) CD86 (an M1 marker) expression on BMDMs gated on F4/80. C) The mRNA expression of M2 markers (CD206, Arg1, and Fizz1) and M1 marker (iNOS) in Raw264.7 cells under different treatment condition upon LPS (100 ng mL^−1^) stimulation. D–F) In vivo efferocytosis assays. WT C57BL/6 mice were received different treatments at 2 h after LPS challenge (10 mg kg^−1^ i.p.), BALF and lung tissues were collected at 24 h after LPS challenge. Representative flow cytometry plots and quantitation of D) M2 type (F4/80 and CD206 double‐positive cells) and E) M1 type (F4/80 and CD86 double‐positive cells) of macrophages in BALF. F) Immunofluorescence staining of lung tissue sections with F4/80 (green) and CD163 (red, an M2 marker) or G) CD86 (red). Scale bar, 100 µm. Data are presented as mean ± SD. *p < 0.05, **p < 0.01, and ***p < 0.001 versus LPS. ^#^ *p* < 0.05, ^##^ *p* < 0.01, and ^###^ *p* < 0.001 versus AOzyme@ACM (one‐way ANOVA with Bonferroni post hoc test).

First, the bone marrow‐derived macrophages (BMDMs) were pre‐cultured with different treatments followed by LPS (100 ng mL^−1^) stimulation and further incubated with apoptotic cells for 2 h, with unstimulated BMDMs as a blank control. We observed that BMDMs treated with AOzyme@ACM effectively promoted phenotype transition evidenced by the stronger MFI of CD206 and weaker MFI of CD86 compared with other groups (**Figure** **5**A,B). The mRNA expression of CD206, arginase‐1 (Arg‐1) and Fizz1, which served as representative markers of M2 macrophages, appeared the highest level and mRNA expression of iNOS (representative M1 marker) appeared the lowest level in AOzyme@ACM treatment (Figure 5C). These results suggested that AOzyme@ACM performed best in promoting macrophage M2 polarization, whether in primary cell or cell line of macrophages. While previous studies have shown that antioxidant itself could lead to anti‐inflammatory phenotype transition in macrophage, ACM‐coated nanozymes behaved better than nanozymes alone, indicating the engagement of the efferocytosis machinery was a significant cause.

Given these results, we further explored whether this synergistic effect occurred in vivo. In this experiment, mice were administered by inhaling Cy5.5‐labeled AOzymes@ACM, AOzyme or PBS, respectively, for 20 min after LPS challenge as described previously. 24 h later, the BALF and lung tissue were collected for further evaluation. The results showed that the proportion of M2 macrophage (F4/80 and CD206 positive cells) was significantly increased in BALF in comparison with PBS or AOzyme treatment, whereas the M1 type (F4/80 and CD86 positive cells) was evidently decreased (Figure 5D,E). These results were consistent with the in vitro findings described above.

Next, we applied immunostaining assay to histologically represent the extensive alleviation of M1 phenotype shift after treatment with AOzyme@ACM. Immunofluorescence staining of lung tissue demonstrated that AOzyme@ACM treatment increased the amount of M2 macrophages, presented as CD163^+^ and F4/80^+^ cells, and reduced the numbers of M1 macrophages, presented as CD86^+^ and F4/80^+^ cells, compared with PBS and AOzyme group (Figure 5F,G), further supporting the role of AOzyme@ACM in eliciting the macrophages polarization toward M2 cells. Based on these results, we demonstrated that ACM coating and ROS inhibition could synergistically contribute to reprogramming inflammatory macrophages toward M2 phenotype and propose a mechanistic model to resolving inflammation by enhancing pro‐resolving phenotype shift in efferocytic macrophages.

### 2.5 Pro‐Efferocytic AOzyme@ACM Alleviated Sepsis‐Related Acute Lung Injury

**Figure 6:**
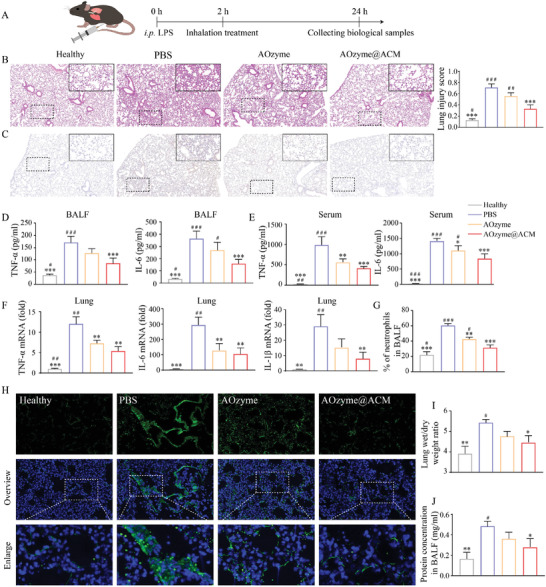
Pro‐efferocytic AOzyme@ACM alleviated sepsis‐related acute lung injury. A) Schematic depicting the animal study design. B) Histopathologic examination using H&E staining. C) Immunohistochemistry of TNF‐*α* expression within lung tissues. D) The level of TNF‐*α* and IL‐6 in BALF and E) serum were measured by ELISA. F) Detection of inflammatory cytokine mRNA (TNF‐*α*, IL‐6, and IL‐1*β*) expression in the lung tissues by RT‐qPCR. G) Neutrophil infiltration in BALF was analyzed by flow cytometry. H) Immunofluorescence staining of mice lung sections for ROS detection (DCFH‐DA, green) and DAPI (blue) in all groups. I) The wet/dry ratio of lung tissue and (J) total protein concentration in BALF were measured. Data are presented as mean ± SD (*n* = 5). *p < 0.05, **p < 0.01, and ***p < 0.001 versus PBS. ^#^ *p* < 0.05, ^##^ *p* < 0.01, and ^###^ *p* < 0.001 versus AOzyme@ACM (one‐way ANOVA with Bonferroni post hoc test).

To investigate the therapeutic effects of AOzyme@ACM and AOzyme, we divided the mice into four groups. Group 1 was healthy mice, groups 2, 3, and 4 were received LPS by intraperitioneal injection (i.p.) and inhaled PBS, AOzyme@ACM, or AOzyme at 2 h post‐LPS challenge, respectively (**Figure** **6**A).

First, we assessed the inflammation status on the histological level. Hematoxylin and eosin (H&E) staining of lung tissue displayed an obvious alleviation in alveolar wall incrassation and alveolar cavity destruction after AOzyme@ACM treatment compared with those treated with AOzym or PBS (Figure 6B). We observed relieved congestion and reduced infiltration of inflammatory cells in AOzyme@ACM treated mice at higher magnification. As mentioned above, prevention of the cytokine storm maybe one of the crucial points to relieve ALI. The levels of TNF‐a, IL‐6, and IL‐1*β* in serum and BALF were examined by ELISA following the treatments. These representative cytokines were significantly reduced in the AOzyme@ACM group compared to the PBS or AOzyme groups, suggesting the therapeutic potential of AOzyme@ACM in the treatment of ALI (Figure 6D,E). Although it has been demonstrated that ROS elimination could suppress the cytokine storm in previous studies, limited therapeutic efficiency was observed compared with AOzyme@ACM in our experimental mouse ALI model, confirming that the cytokine storm was potently calmed by ACM coating‐dependent efferocytic resolution of inflammation. Besides, immunohistochemistry was used to measure the level of TNF‐*α* in lung and AOzyme@ACM administration exhibited the greatest reduction on TNF‐*α* expression (Figure 6C). To assess the inflammation status more comprehensively, we measured the mRNA expression of proinflammatory cytokines in lung tissue. We observed decreased mRNA levels of TNF‐*α*, IL‐6, and IL‐1*β* in mice treated with AOzyme@ACM compared to mice treated with AOzyme (Figure 6F).

Neutrophil infiltration and activation within the lung are important factors in the pathogenesis of sepsis related ALI, and we subsequently characterized the inhibition of neutrophil accumulation in BALF by flow cytometry. The total percentage of Ly6G^+^ cells in BALF was reduced significantly in the mice receiving the AOzyme@ACM compared to the AOzyme alone, exhibiting a more rapid resolution of airway neutrophilia (Figure 6G; Figure S19, Supporting Information). As the production of ROS can characterize the severity of lung injury, lung tissue was stained by DCFH‐DA for ROS detection. It was found that AOzyme@ACM treatment could significantly inhibit the generation of ROS during acute pneumonia, while AOzyme treatment showed a limited ROS‐scavenging capability (Figure 6H). In addition, we measured the lung wet/dry weight ratios and proteinaceous exudates to examine lung edema (Figure 6I,J). The prominent edema and vascular leaking in the untreated group were significantly ameliorated by AOzyme@ACM inhalation, again confirming histological examination.

## 3 Conclusions

Efferocytosis, an immunologically silent process, is a prerequisite for resolution of inflammatory lung injury. Inspired by the inherent positive feedback between “eat me” signal recognition and amplified removal of dying cells, we developed a strategy based on correcting efferocytosis defect through combing ACM coating and antioxidant nanozyme. We found that AOzyme@ACM not only reprogrammed pro‐inflammatory macrophages toward a pro‐resolving phenotype, but also suppressed the activation of NF‐*κ*B signal, both of which mainly benefited from the enhanced efferocytosis ability. In vivo results showed that mice in AOzyme@ACM treatment group exhibited effective apoptotic cells clearance, suppressed inflammatory cytokines production, reduced inflammatory cell infiltration, and lung edema. Our findings raise the intriguing possibility of ACM coating and antioxidant nanozyme‐based therapy through focusing on restoring impaired efferocytosis to orchestrate homeostasis in ALI, even a broad platform for inflammation resolution.

## Conflict of Interest

The authors declare no conflict of interest.

## Supporting information

Supporting Information

## Data Availability

The data that support the findings of this study are available from the corresponding author upon reasonable request.;
