# Supplementary material for: Inhaled Pro‐Efferocytic Nanozymes Promote Resolution of Acute Lung Injury
Source: Adv Sci (Weinh). 2022 Jul 20;9(26):2201696. doi: 10.1002/advs.202201696 (PMC9475552; doi:10.1002/advs.202201696)
Supplement: Supplementary file 1 — Supporting Information [file ADVS-9-2201696-s001.pdf]

ADVANCED SCIENCE
Open Access

Supporting Information

for *Adv. Sci.*, DOI 10.1002/advs.202201696

Inhaled Pro-Efferocytic Nanozymes Promote Resolution of Acute Lung Injury

Haiying Ji, Chengmi Zhang, Fengying Xu, Qianyun Mao, Ran Xia, Muqiao Chen, Wei Wang, Shunan Lv, Weiwei Li and Xueyin Shi**

Supplementary information

Inhaled Pro-efferocytic Nanozymes Promote Resolution of Acute Lung Injury

Haiying Ji[#], Chengmi Zhang[#], Fengying Xu[#], Qianyun Mao, Ran Xia, Muqiao Cheng, Wei Wang, Shunan Lv, Weiwei Li, Xueyin Shi**

H. Ji, C. Zhang, R. Xia, M. Cheng, W. Wang, S. Lv, W. Li, Prof. X. Shi

Department of Anesthesiology and Intensive Care Unit, Xinhua Hospital, School of Medicine, Shanghai Jiaotong University, Shanghai 200092, China.

*E-mail: shixueyin@xinhuamed.com.cn; luckyliweiwei@gmail.com

F. Xu

Department of Anesthesiology, No. 971 Hospital of People's Liberation Army Navy, Qingdao 266000, China.

Q. Mao

Department of Endocrinology, Xinhua Hospital, School of Medicine, Shanghai Jiaotong University, Shanghai 200092, China.

[#] These authors contributed equally to this work.

Experimental Section

Materials: Tetraethyl orthosilicate (TEOS), poly (allylamine hydrochloride) (PAH, MW≈15,000), 2′,7′-dichlorofluorescein diacetate (DCFH-DA), and (3-Aminopropyl) triethoxysilane (APTES) were purchased from Sigma-Aldrich (USA). Ethyl alcohol, polyacrylic acid (PAA, MW≈1,800), hydrogen peroxide (H_2O_2, 30% aqueous solution), Potassium permanganate ($KMnO_4$), Sodium Carbonate (Na_2CO_3) were purchased from Shanghai (China) Reagent Company. Polycarbonate membrane (200 nm) were purchased from Avanti. Superoxide anion ($O_2^{\bullet-}$) assay kit and Glutathione (GSH) were purchased from NanJing JianCheng Bioengineering Institute. Annexin V-FITC/PI Apoptosis Detection Kit, Hydrogen Peroxide Assay Kit, Total Superoxide Dismutase Assay Kit with NBT, 4',6-diamidino-2-phenylindole (DAPI), Fast Silver Stain Kit, Mitochondrial Membrane Potential Assay Kit with TMRE, dihydroethidium and Cell Counting Kit-8 (CCK-8) were purchased from Shanghai Beyotime Co, Ltd. PEG polymers were obtained from JiaXing BoMei, China. Cy5.5 NHS ester, Methylene blue (MB) and FITC were purchased from Aladdin Co, Ltd. RPMI-1640 medium, Fetal bovine serum (FBS) and penicillin-streptomycin with 0.25% trypsin-EDTA were purchased from Gibco Corp. Phosphate-buffered saline (PBS)

were purchased from Hyclone Corp. FITC-anti-CD206 (#141704), APC-anti-F4/80 (#123116) and anti-CD16/32 (#101319) antibodies were supplied by Biolegend (USA). FITC-anti-Gr-1(#11-5931-81), FITC-anti-Ly6G (#11-5931-81) antibody, CellTracker™ Red CMTPX, PageRuler prestained Protein (#26616), Hoescht 33342 and CD47 antibody (#MA5-11895) were supplied by Thermo Fisher Scientific (USA). FITC-anti-CD86 (#553691) was from BD Bioscience. P-p65 antibody (#3033S), p65 antibody (#8242S), β-actin antibody (#3700S) Na, K-ATPase antibody (#3010S) and GAPDH antibody (#5174) were supplied by Cell Signaling Technology. F4/80 antibody (#GB113373) was purchased from Servicebio (China). ECL detection reagent was from Millipore Corporation (USA). Lipopolysaccharides (LPS, from Escherichia coli O111:B4) were purchased from Sigma-Aldrich (USA). Mouse macrophage colony-stimulating factor (M-CSF) was obtained from PeproTech (USA). Nebulizer was purchased form Feellife Health Inc (China).

Characterization: Dynamic light scattering (DLS) measurements were carried out at 298.0 K using Zetasizer Nano-ZS (Malvern, UK) equipped with standard 633 nm laser. JEM-2010 transmission electron microscopy (TEM) was used to characterize the morphology of nanoparticle. High-angle annular dark-field scanning TEM (HAADF-STEM) image and element mapping were obtained by Titan Themis 60-300 G2. XPS analysis was conducted by the X-ray Photoelectron Spectrometer (Thermo Kalpha). UV-vis spectra of different samples were recorded by the UV-vis spectrophotometry Lambda 35 (Perkin-Elmer).

Synthesis of $HMnO_2$: (1) SiO_2 nanoparticles (NPs) were prepared according to the Stöber method.[1] SiO_2 NPs were functionalized with amino group. 200 mg of SiO_2 NPs was dispersed in a mixture of deionized water (DIW) and ethanol, and with 500 µl of APTES added for the reaction system. The mixture was stirred at room temperature for 1 hour and heated at 80 °C for 2 h. SiO_2-NH_2 NPs were obtained after a washing process with ethanol.[2] (2) The SiO_2-NH_2 NPs serve as a template to construct the hollow nanostructures.[3] Briefly, 200 mg of SiO_2 nanospheres was dispersed in 100 ml of DIW and the solution was sonicated and stirring for 10 min. $KMnO_4$ (20 ml, 20 mg/ml) was then added into the solution drop by drop under stirring for 12 h. After that, the resultant SiO_2@MnO_2 NPs were collected by centrifugation and washed three times with water. (3) The as-prepared SiO_2@MnO_2 was dissolved in Na_2CO_3 aqueous solution (2 M) at 60°C for 6 h. The $HMnO_2$ NPs were obtained by centrifugation and washed three times with DIW.

Synthesis of Cu NPs: In a typical preparation process, $CuCl_2 \cdot 2H_2O$ aqueous solution was prepared by dissolving $CuCl_2 \cdot 2H_2O$ (10 mM) in 50 ml DIW.[4] Subsequently, $CuCl_2 \cdot 2H_2O$ aqueous solution was heated to 80°C in an oil bath with magnetic stirring. A 50 ml L-ascorbic acid aqueous (1 M) solution was added dropwise into the flask while stirring. The mixture was kept at 80 °C for 24 h. The resulting dispersion was centrifuged at 8,000 rpm for 15 min. The supernatant was dialyzed for three days to obtain Cu NPs.

Synthesis of AOzyme: 10 ml $HMnO_2$ solution (2 mg/ml) was then added to 20 ml PAH solution (5 mg/ml) under ultrasonication. After 2 h of stirring, the above solution was centrifuged and washed with water. The obtained $HMnO_2$-NH_2 NPs were added into Cu NPs aqueous solution (20 ml, 0.1 mg/ml) under ultrasonic agitation. After stirring for 12 h, the prepared $HMnO_2$-Cu NPs was collected by centrifugation and washed with water for three times.

To obtain $HMnO_2$-Cu-PEG (AOzyme) NPs, 10 ml $HMnO_2$-Cu solution (2 mg/ml) was then added to 20 ml PAH solution (5 mg/ml) under ultrasonication. After 2 h of stirring, the above solution was centrifuged and washed with water. 10 ml $HMnO_2$-Cu-PAH solution (2 mg/ml) was then added to 20 ml PAA solution (5 mg/ml) under ultrasonication. After 2 h of stirring, the above solution was centrifuged and washed with water, before it was mixed with 50 mg mPEG-5K-NH_2 under ultrasonication for 30 min. After adding 15 mg EDC and stirring for 12 h, the prepared $HMnO_2$-Cu-PEG was collected by centrifugation and washed with water for three times and named AOzyme.

ICP-AES analysis of copper content in $HMnO_2$-Cu composite nanoparticles: The $HMnO_2$-Cu nanoparticles were dissolved using aqua regia and subsequently diluted to the test concentration. Subsequently, the mass fraction of elemental copper was characterized by inductively coupled plasma atomic emission spectrometry (ICP-AES).

Cell Culture: Murine Raw264.7 macrophages (American Type Culture Collection, ATCC), the human acute promyelocytic leukemia cell line PLB-985 cells (ATCC) and the human leukemia Jurkat T cells (ATCC) were cultured in RPMI 1640 supplemented with 10% FBS plus penicillin streptomycin-glutamine at 37°C in 5% CO_2. The PLB-985 cells were seeded at 10^6 cells/ml in the above-mentioned cell medium supplemented with 1.25% DMSO for 5 days to allow for neutrophil-like differentiation, as described.[5]

Primary cell isolation and culture: Bone marrow-derived macrophages (BMDMs) were extracted from the femur and tibias of WT mice (6-8 weeks) by flushing sterile PBS to the end of the bone using a syringe. The extract was centrifuged at 2,000 rpm for 5 min and then the pellet was resuspended in RPMI 1640 supplemented with 10% FBS, 1% antibiotics, containing mouse M-CSF (20 ng/ml) to prepare cell suspensions at 1×10^6 cells/ml. Media was changed on day 3 and 5 and Cells were used for experiments at day 6 of culture.

To isolate mouse peritoneal macrophages (PMs), mice were sacrificed and the peritoneal cavity was exposed. Then 5 ml of ice-cold sterile RPMI 1640 medium was injected into the peritoneal cavity. After gently massaged, the injected fluid was collected as possible. The collected cell suspension was centrifuged at 2,000 rpm for 5 min and then the pellet was resuspended in culture medium mentioned earlier. After 4 h of incubation, unattached cells were removed, and the adherent cells were used for

further experiments.

Induction of apoptosis: Differentiated PLB-985 cells and Jurkat T cells were induced apoptosis by exposure to UV irradiation at 312 nm/254 nm for 20 min and were cultured for a further 3 h before use.

Synthesis of AOzyme@ACM and AOzyme@NCM: To achieve the apoptotic cell membrane (ACM) or normal cell membrane (NCM), differentiated PLB-985 cells, which were induced to apoptosis or not, were first dispersed in homogenization medium containing 0.25 M sucrose, 1 mM EDTA, 20 mM HEPES, and protease inhibitor cocktail (pH 7.4). The mixture was sonicated in an ice bath using ultrasonic crusher (on = 5 s, off = 5 s, 3 min) and then centrifuged (2,000 rpm, 10 min, 4°C) to remove the precipitate. The gathered supernatant was further centrifuged (15,000 rpm, 20 min, 4°C) to collect membranes. For membrane coating, after $HMnO_2$-Cu NPs and membranes (NPs-to-membrane weight ratio of 1:1) were dispersed in saline and extruded ten times with 200 nm porous polycarbonate membranes by an extruder. Coating completeness was confirmed by studying nanoparticle stability in 1× PBS or RPMI-1640 containing 10% FBS.[6]

$O_2^{\bullet-}$ scavenging activity of NPs: The superoxide anion ($O_2^{\bullet-}$) scavenging activity was assessed using a superoxide anion assay kit according to the manufacturer’s instructions. [4] The kit simulates the reaction system of xanthine and xanthine oxygenase in the body, generating $O_2^{\bullet-}$, adding electron transferring substance and color developer to make the reaction system appear purple-red, and the relative content of $O_2^{\bullet-}$ can be obtained by measuring the absorbance with spectrophotometer. Vitamin C (Vc) can efficiently scavenge $O_2^{\bullet-}$, so the $O_2^{\bullet-}$ scavenging rate of the Vc group was set at 100%, and the formula for $O_2^{\bullet-}$ scavenging efficiency can be obtained as follows:

$$O_2^{\bullet-} \text{ scavenging rates} = \left(1 - \frac{Abs_{Tset} - Abs_{Vc}}{Abs_{O_2^{\bullet-}} - Abs_{Vc}}\right) \times 100\%$$

Different concentrations of NPs (Cu NPs 2.2 μg/ml；$HMnO_2$ 47.8 μg/ml；$HMnO_2$-Cu 50 μg/ml; AOzyme; AOzyme@ACM) were added to the working solution. After incubating for half an hour, the absorption at 550 nm was measured to assess the $O_2^{\bullet-}$ scavenging activity.

H_2O_2 scavenging activity of NPs: H_2O_2 scavenging capacity of NPs was tested by the H_2O_2 Detection Kit.[4] The kit produced trivalent iron ions by oxidizing divalent iron ions with H_2O_2, and then reacts with xylenol orange to form a purple product which had strong absorption at about 550 nm. The standard curve can be obtained by using the ELISA Reader to detect the optical density value at 550 nm after the reaction of the kit with the hydrogen peroxide standard. The representative standard curve of H_2O_2 linearly fitted between absorption intensity vs H_2O_2 concentration was shown in Figure S12.

Various concentrations of NPs were incubated with 2 mM H_2O_2 for 0.5 h, respectively. The nanoparticles were removed by centrifugation at the end of the reaction, and the concentration of the remaining H_2O_2 was determined according to the production instructions. The value of H_2O_2 scavenging rates were obtained by the following equation:

$$H_2O_2 \text{ scavenging rates} = \frac{C_0\text{-}C}{C_0} \times 100\%$$

.

•OH scavenging activity of NPs: Methylene blue (MB) can be degraded by •OH but not by H_2O_2. Therefore, the amount of •OH produced can be reflected by detecting the change in the concentration of MB before and after the reaction.[7] The ·OH-induced MB degradation was monitored by the absorbance change at 665 nm (Figure S13). The value of MB degradation rates (DR) was obtained by the following equation:

$$\text{MB degradation rates} = \frac{C_0\text{-}C}{C_0} \times 100\%$$

In detail, the working test solutions containing 5 μg/ml MB, 2 mM H_2O_2, 1 mM $FeSO_4$ and different concentrations of NPs in PBS buffer (0.05 M, pH=6) were prepared in the dark and rest for 30 min. Then, the absorbance peak in 665 nm of the solution was monitored with a UV-vis spectroscopy after centrifugation to remove the nanoparticles. And the value of •OH scavenging rates were obtained by the following equation:

$$\bullet\text{OH scavenging rates} = \frac{DR_{Fe^{2+}+H_2O_2} - DR_{Test}}{DR_{Fe^{2+}+H_2O_2}} \times 100\% = \frac{C_{Test} - C_{Fe^{2+}+H_2O_2}}{C_0 - C_{Fe^{2+}+H_2O_2}} \times 100\%$$

Cytotoxicity of NPs: Raw264.7 cells were seeded into 96-well plates at a density of 10000 cells per well and cultured overnight. Then the culture media were replaced by fresh medium containing NPs at varied concentrations of 0,6.25,12.5,25, 50, 100, 200 and 400 μg/ml. After further incubation for 24 h, the culture media were replaced by FBS-free medium containing 10% CCK-8. After further co-incubation for 1 h, the cell proliferation was determined using the microplate reader by comparing the absorbance at 450 nm to the control group.

Cellular uptake assay: The NPs uptake into the Raw264.7 cells was evaluated by fluorescence microscopy and flow cytometry. For confocal fluorescence microscope, the Raw264.7 cells were cultured on 14 mm glass coverslips in plates with a culture medium for 12 h before experiment. Then, the cells were pre-incubated to fresh medium containing with Cy5.5-labeled NPs (50 μg/ml) after exposed to LPS (100 ng/ml). After incubating with NPs for 1 h and rinsed three times by PBS, cells were fixed with 4% paraformaldehyde for 10 min and washed again with PBS. Cell nuclei were stained with Hoescht 33342 for 10 min and observed under the confocal laser scanning microscope (CLSM). For flow cytometry, the Raw264.7 cells were cultured

in 24-well plates and then treated with Cy5.5-labeled NPs (50 μg/ml) under the same conditions described above. After that, the cells were washed with PBS to remove free NPs and collected by centrifugation at 1,000 rpm. The intensity of intracellular Cy5.5 was determined by flowjo.

Protecting cells from H_2O_2 or Fenton's reagent-induced oxidative stress: Raw264.7 cells were seeded in 96-well plates at a density of 10^4 cells/well overnight. Then the cells were incubated with multiple NPs for 0.5 h, followed by H_2O_2 (0.5 mM) or Fenton's reagent (0.5 mM of H_2O_2 and 50 μM of $FeSO_4$) treatment for 24 h. Cell viability was evaluated by CCK-8 assay. Furthermore, cells seeded in 24-well plates were stained with Annexin V-FITC/PI apoptosis detection kit to detect the ratio of apoptotic cells. Furthermore, mitochondrial membrane potential kit was used to assess mitochondrial damage.

Intracellular ROS evaluation: Raw264.7 cells were seeded (1×10^5 cells in 1 ml RPMI-1640) in the 24-well cell culture plate and allowed to adhere overnight. The culture media were then replaced by new medium containing indicated NPs and divided as the following groups: 1) control; 2) LPS; 3) LPS+Cu NPs; 4) LPS+$HMnO_2$; 5) LPS+AOzyme; 6) LPS+AOzyme@NCM 7) LPS+AOzyme@ACM. After incubation for 2 h, the culture media were replaced by 1 ml DCFH-DA (10 μM) and incubated for 30 min. The cells were washed with PBS three times and the intracellular ROS was evaluated by flow cytometry analysis. The intracellular $O_2^{\bullet-}$ detected by the superoxide anion detection kit and fluorescence intensity was measured using confocal microscopy to show the content of superoxide anion free radical.

In vitro efferocytosis assays: Labeled apoptotic Jurkat T cells were added to macrophages (Raw264.7 cells，BMDMs or PMs) at a 5:1 ratio (apoptotic cell to phagocytes) in the presence of PBS or NPs and incubated at 37°C in 10% CO_2 for 2 h. In all cases, macrophages were pretreated with NPs for 0.5 h at the indicated concentrations before LPS (100 ng/ml) treatment. Macrophages were washed with cold PBS to remove suspended cells and analyzed by flow cytometry or other measurements. For confocal imaging，apoptotic Jurkat T cells were labelled with Cell Tracker Red CMPTX dyes (2 μM) and efferocytosis was determined by visual inspection of samples and was expressed as the Efferocytosis index (PI). For flow cytometry, apoptotic Jurkat T cells were labelled with carboxyfluorescein succinimidyl ester (CFSE, 2.5 μM) and efferocytic activity was evaluated as the percentage of F4/80-labeled macrophages that phagocytosed CFSE-labeled ACs.

Protein detection and western blot: The protein contents of the ACM, NCM, AOzyme@NCM and AOzyme@ACM were first determined by sodium dodecyl sulfate-polyacrylamide gel electrophoresis (SDS-PAGE). The membrane proteins were extracted from the normal or apoptotic differentiated PLB-985 cells, AOzyme@NCM and AOzyme@ACM with the RIPA lysis buffer and further

measured by electrophoresis assay and dyed with silver staining.

For NF-κB activation detection, Raw264.7 cell lysates were separated on 10% SDS-PAGE. For CD47 expression detection, protein sample extracted from lysates of membranes and coating NPs were separated on 10% SDS-PAGE. Separated proteins were electrophoretically transferred onto PVDF membranes and blocked in TBS-0.1% tween with 5% non-fat dried milk. The membranes were probed with specific antibodies to phospho-p65 at serine 536, p65 and GAPDH at 4 °C overnight. Finally, the PVDF membranes were further incubated with horseradish peroxidase conjugated secondary antibody and visualized by chemiluminescence (ECL).

Gene expression analysis by reverse transcription-quantitative PCR(RT-qPCR): Total RNA was isolated from Raw264.7 cells or lung tissue using TRIzol (TAKARA, Japan) following the manufacturer's instruction The cDNA thus obtained using PrimeScript RT reagent Kit (#RR036; TAKARA) and PCR was performed using TB Green™ Premix Ex Taq™ (Tli RNaseH Plus) (#RR420A; TAKARA). *GAPDH* expression was used as an internal control. QuantStudio™3 System (Applied Biosystems) was used for quantitative PCR analysis. Relative gene expression was analyzed by ΔΔCt method and primer sequences are shown in Table S1.

Animals: Six- to eight-week-old wild-type C57BL/6 mice were purchased from Shanghai Sippr-BK Laboratory Animal Co. Ltd. The animals were fed under a specific pathogen-free environment in the Shanghai Xinhua Hospital animal laboratory. All animal experiments were conducted under the rules approved by the Ethics Committee of the Xinhua Hospital, Shanghai Jiaotong University School of Medicine.

Inhalation of NPs and In vivo distribution: Aerosol was generated via a medical-grade nebulizer. Mice were placed in the chamber to inhale Cy5.5 labeled CM-coated NP for 30 min. The lungs were dissected at 6 h (n = 3/time) for *ex vivo* imaging and the biodistribution of Cy5.5 labeled CM-coated NP in the lungs was monitored using IVIS Spectrum In Vivo Imaging System (PerkinElmer, Waltham, MA, USA). Filters allowing excitation at 560 nm and collection of emission at 590 nm were used to obtain ideal images.

Inhibition of Acute lung injury in vivo: All the animals were randomly assigned to the experimental groups. Then 150 μl of PBS containing of LPS from Escherichia coli (Sigma-Aldrich) was intraperitoneally administrated (10 mg/kg). At 2 h post the challenge, the mice received inhalation of PBS or PBS containing NPs for 20 min, then killed at 24 h post LPS treatment. The trachea was separated and being made a transverse incision for inserting a lavage catheter. BALF was carefully collected with 1 ml of cold PBS containing 2% FBS three times. The cell pellets obtained after centrifugation were resuspended in 1 ml PBS.

For Flow cytometry analysis, after red blood cell lysis and blocking Fcγ receptors with mouse anti-CD16/CD32, cells were incubated with the following antibodies

according to the manufacturer's instructions: anti-CD86, anti-CD206 and anti-F4/80 antibodies were used for fluorescent staining.

To assess the efferocytosis of macrophage in BALF, cells from BALF were immunostained for F4/80 and intracellular Gr-1 and subjected to flow cytometric analysis. Permeabilization is needed to detect Gr-1, indicating that the Gr-1 is intracellular, a marker of macrophage engulfment of neutrophils.

For proinflammatory cytokines measurement，The level of IL-6 and TNF-α in the BALF supernatant were determined by commercially available ELISA Kit according to manufacturer's guidelines. BALF sample protein concentration was used as an indicator of blood-pulmonary epithelial cell barrier integrity. The total proteins in the BALF supernatant were measured by a bicinchoninic acid (BCA) detecting assay.

At 24 h post the LPS challenge, all mice were killed, lung tissues were harvested. The lung tissues were fixed in 4% paraformaldehyde and embedded in paraffin. After routine processing, sections were stained with either hematoxylin and eosin (H&E) and/or immunohistochemical staining, or processed for immunostaining. To evaluate the lung injury via H&E staining, five independent random lung fields were evaluated per mouse for neutrophils in alveolar spaces, neutrophils in interstitial spaces, hyaline membranes, proteinaceous debris filling the airspaces, and alveolar septal thickening, and weighed according to the official American Thoracic Society workshop report on features and measurements of experimental ALI in animals.[8] The resulting injury score is a continuous value between 0 and 1.

For immunohistochemical staining, the sections were incubated in 3% bovine albumin serum (BSA) solution and subsequently were probed overnight at 4 ℃ with primary antibodies against TNF-α. Horseradish peroxidase-conjugated secondary antibodies and 3,3′-duanunibenzidine were used to visualize antigens according to the manufacturer's protocol.

For immunofluorescence staining, paraffin-embedded lung tissue sections were blocked with PBS containing 3% BSA, permeabilized with PBS/Triton 0.01%, and incubated with the anti-F4/80 and anti-86 or anti-CD163 antibodies, and then with species-specific secondary antibodies coupled with fluorescence dyes. Nucleus was stained using DAPI.

Statistical analysis：Quantitative data are indicated as mean ± S.D. Statistical analyses were performed on SSPS Statistics 26. 2-tailed Student's t test was performed for comparisons between 2 groups under identical conditions. Multiple group comparisons were performed by one-way ANOVA followed by Bonferroni's multiple comparisons test, *($^{\#}$), **($^{\#\#}$), and ***($^{\#\#\#}$) indicate $P < 0.05$, $P < 0.01$, and $P < 0.001$, respectively.

Table S1. Primers for quantitative RT-qPCR.

Primer	Forward (5'-3')	Reverse (5'-3')
IL-1β	GAAATGCCACCTTTTGACAGTG	TGGATGCTCTCATCAGGACAG
IL-6	TAGTCCTTCCTACCCCAATTTCC	TTGGTCCTTAGCCACTCCTTC
TNF-α	CAGGCGGTGCCTATGTCTC	CGATCACCCCGAAGTTCAGTAG
Arg1	ACATTGGCTTGCGAGACGTA	ATCACCTTGCCAATCCCCAG
CD206	CTCTGTTCAGCTATTGGACGC	TGGCACTCCCAAACATAATTTGA
Fizz1	ACTTTGATGGCCTCAACCTG	AATGATTCCTGCTCCTGTGG
CD36	GCTGGATTAGTGGTTAGGAGTCA	GAGAGGCGGGCATAGTATCA
iNOS	CAGATCGAGCCCTGGAAGAC	CTGGTCCATGCAGACAACCT

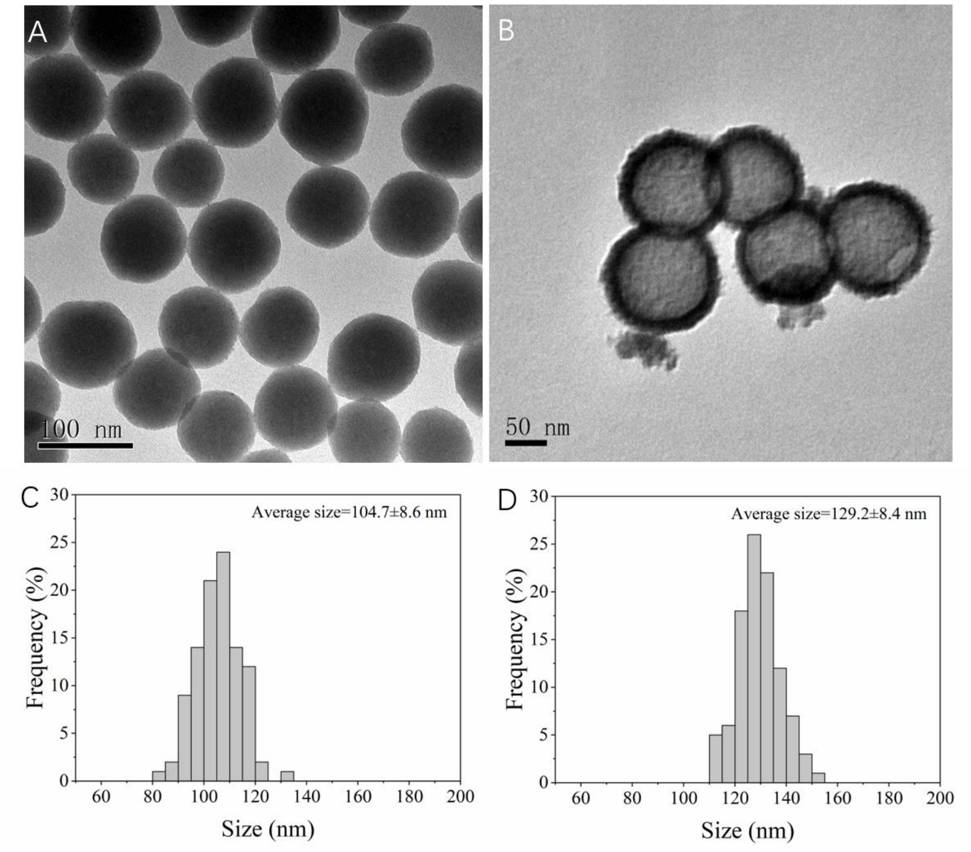

Figure S1. TEM images of (A) SiO_2 and (B) $HMnO_2$ nanoparticles. The size distribution profile of (C) SiO_2 and (D) $HMnO_2$ nanoparticles based on their TEM images (n=100).

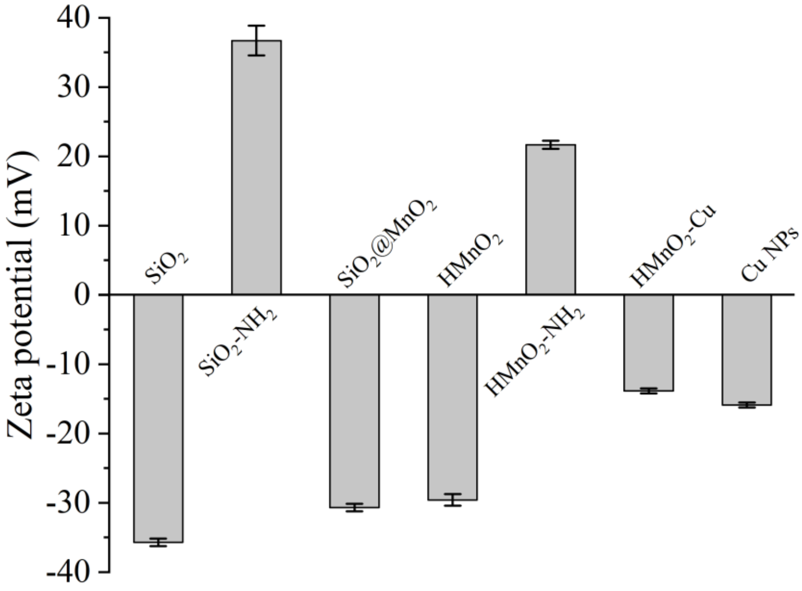

Figure S2. The changes of zeta potential of the nanoparticles obtained under different synthetic conditions. Date are presented as means ± SD (n = 3).

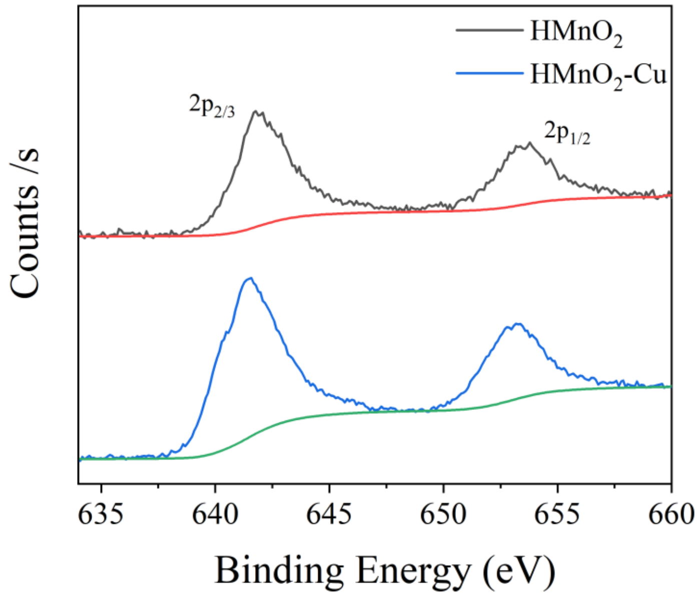

Figure S3. XPS Mn2p spectra of $HMnO_2$ and $HMnO_2$-Cu NPs.

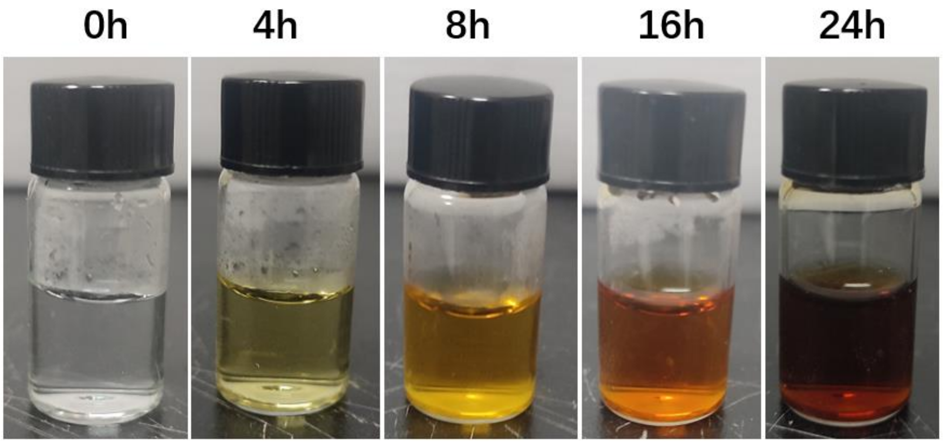

Figure S4. Photographs of dispersion's color change over time in the synthesis of Cu NPs.

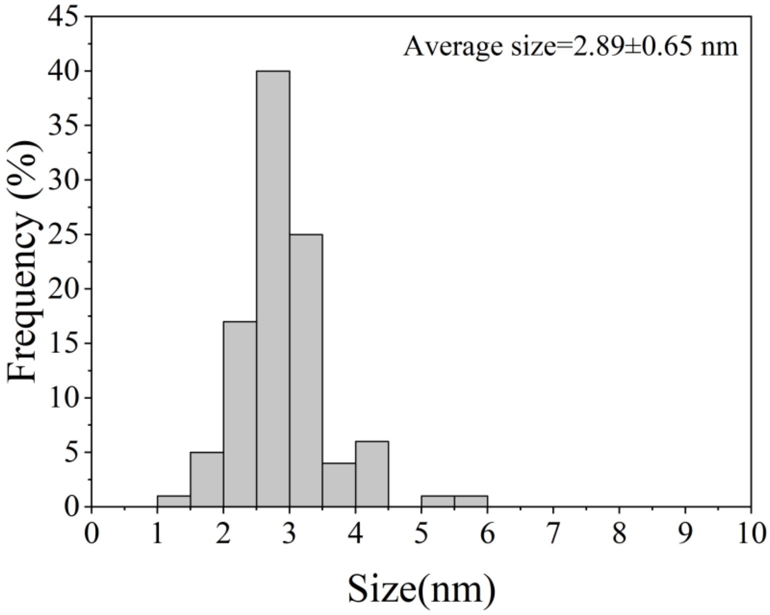

Figure S5. The size distribution profile of Cu NPs based on Figure 1 TEM images (n=100).

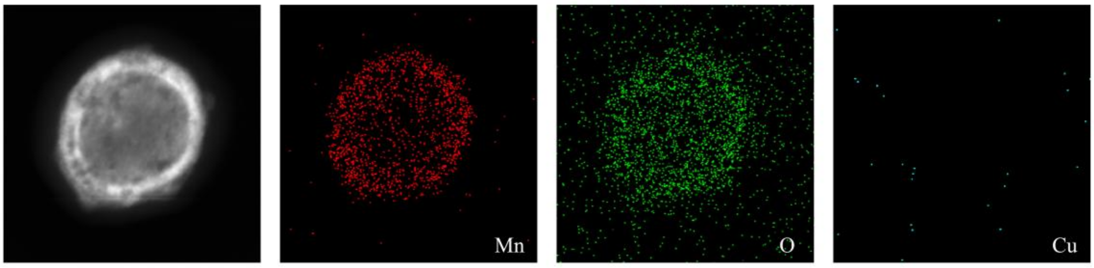

Figure S6. STEM image of the as-prepared $HMnO_2$ NPs, showing the element distribution of Mn, O and Cu.

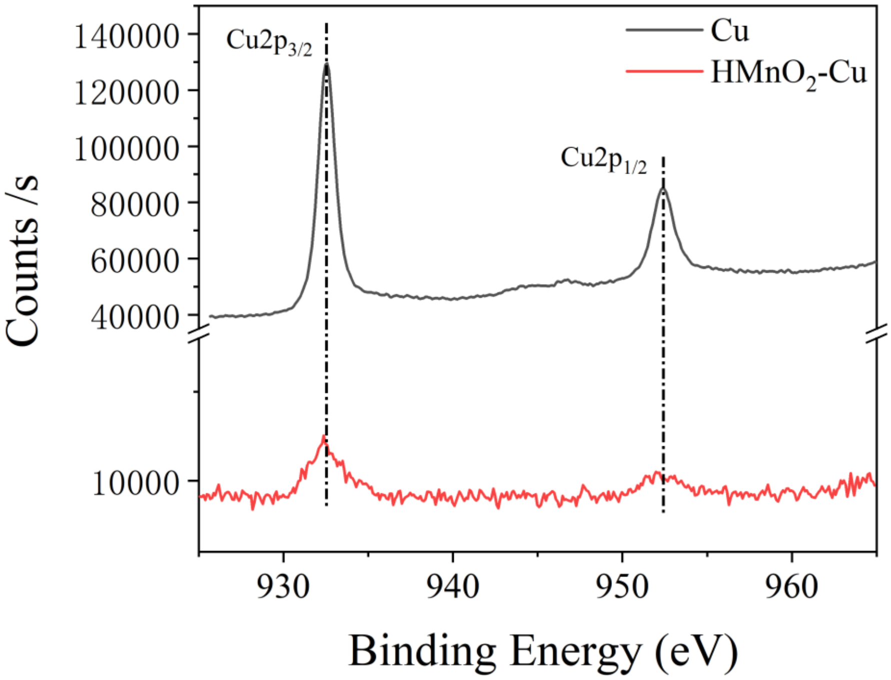

Figure S7. XPS Cu2p spectra of Cu NPs and $HMnO_2$-Cu composite.

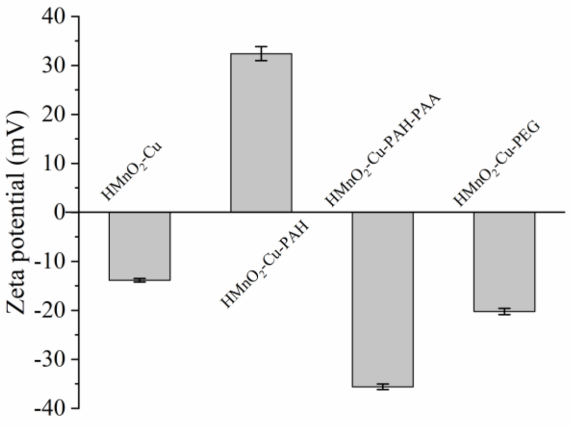

Figure S8. The changes of zeta potential of nanoparticles during the PEG modification. Date are presented as means ± SD (n = 3).

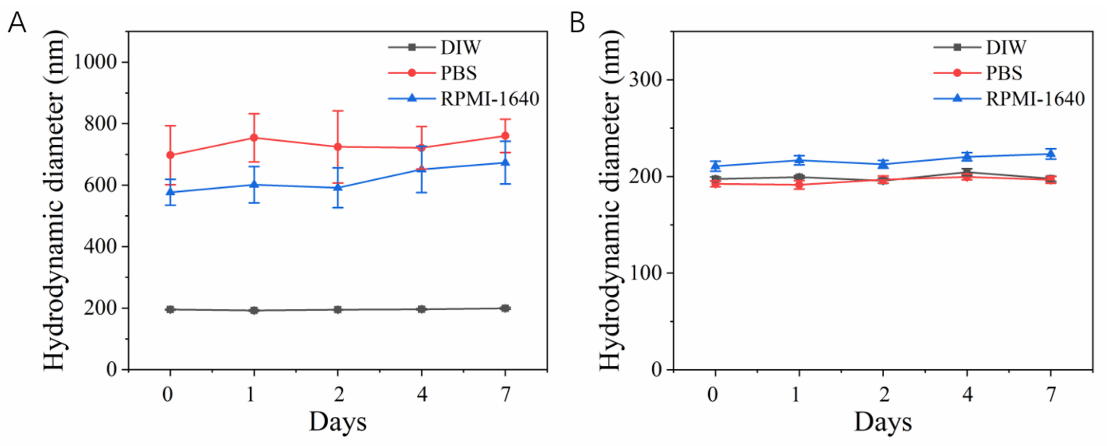

Figure S9. Time-dependent DLS size variations of (A) $HMnO_2$-Cu NPs and (B) $HMnO_2$-Cu-PEG NPs (AOzyme) over time recorded on multiple samples in water, PBS and RPMI-1640 medium for 7 days as determined by the DLS measurement. Date are presented as means ± SD (n = 3).

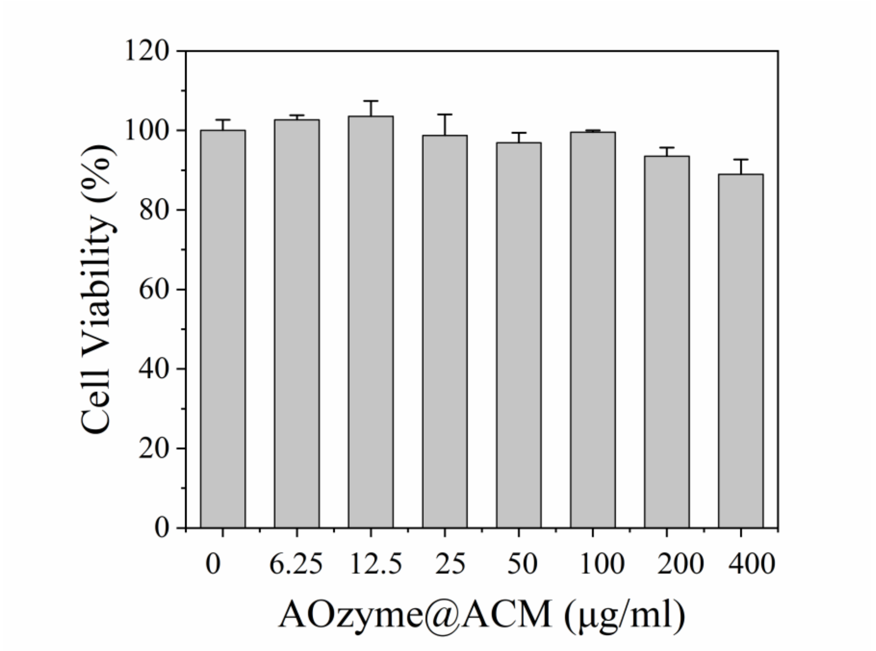

Figure S10. Cell viability of Raw264.7 cells treated with various concentrations of

AOzyme@ACM for 24h. Date are presented as means ± SD (n = 3).

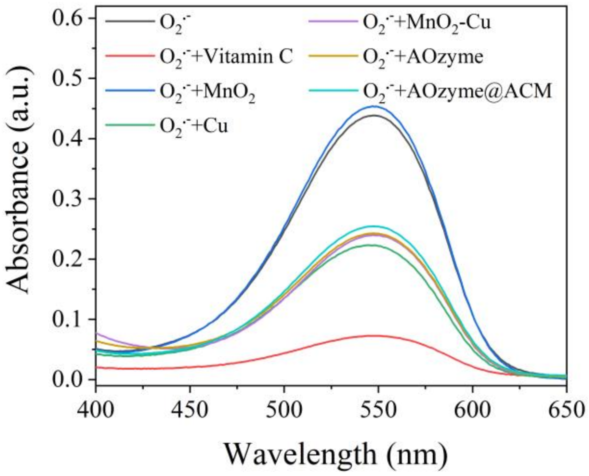

Figure S11. UV-Vis absorption spectra of various nanoparticles for superoxide anion radical scavenging experiments.

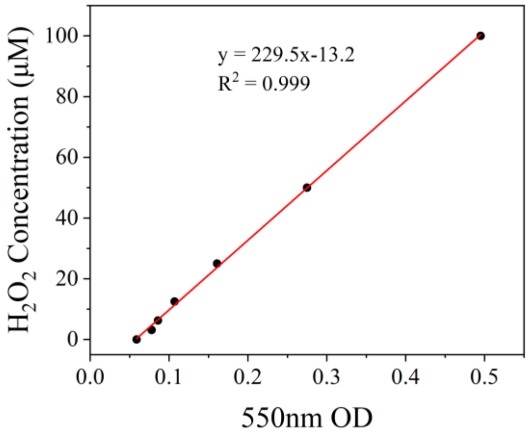

Figure S12. The standard curve of H_2O_2 linearly fitted between absorption intensity vs H_2O_2 concentration.

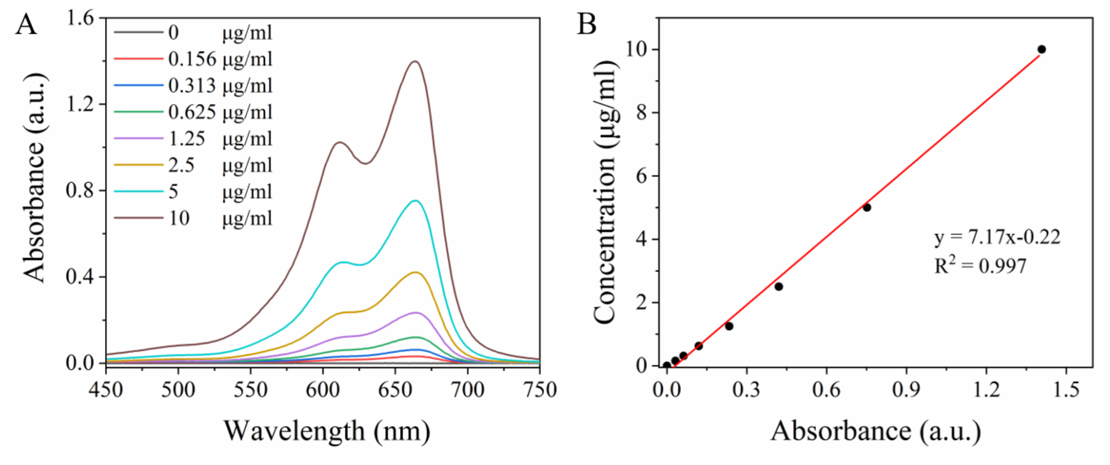

Figure S13. (A) UV-Vis absorption spectra of MB at different concentrations. (B) The standard curve of MB linearly fitted between absorption intensity vs MB concentration.

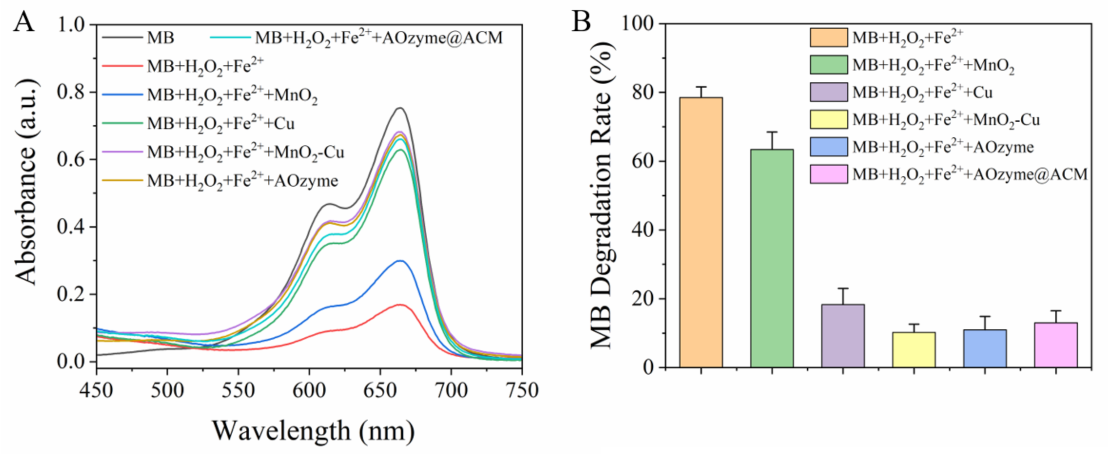

Figure S14. (A) UV-Vis absorption spectra of MB after degradation by the Fe^{2+}-mediated Fenton-like reaction with the addition of different nanoparticles. (B) Degradation rate of MB under different conditions. Date are presented as means ± SD (n = 3).

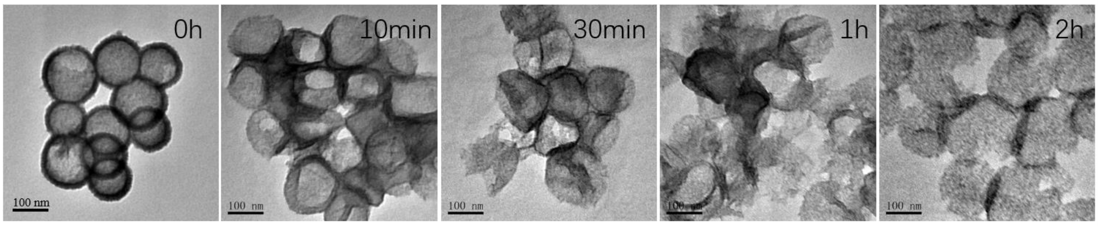

Figure S15. TEM image of $HMnO_2$-Cu-PEG nanocomposites (AOzyme) after incubated in PBS at pH6.5 + 100 μM H_2O_2 for 2h.

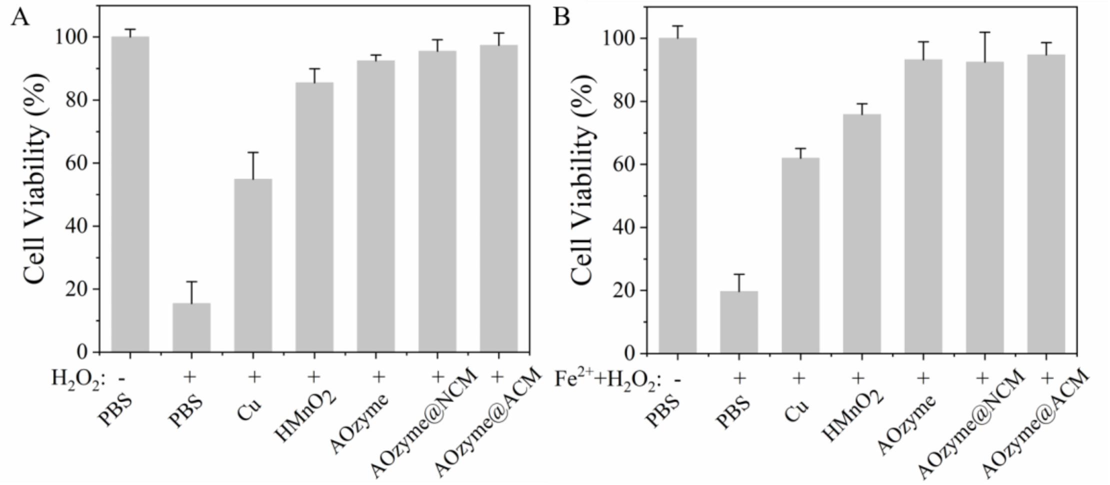

Figure S16. Cell viability of Raw264.7 cells treated by Cu NPs, $HMnO_2$, AOzyme, AOzyme@NCM and AOzyme@ACM in presence of (A) 500 μM H_2O_2 or (B) Fenton reagent (100 μM $FeSO_4$ + 500 μM H_2O_2). Date are presented as means ± SD (n = 3).

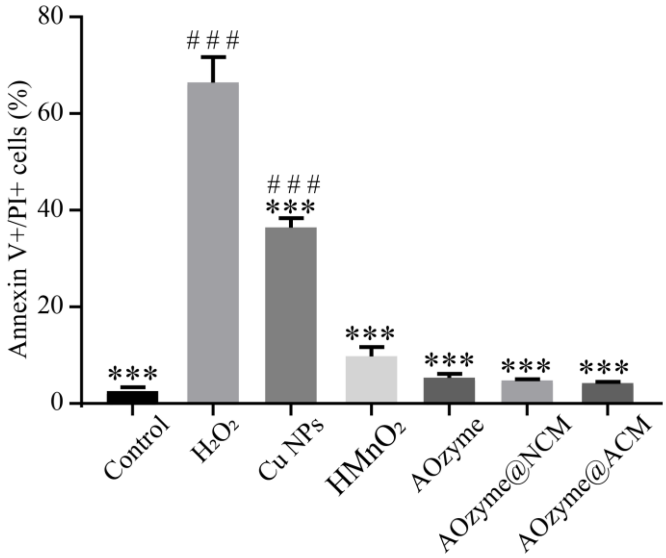

Figure S17. Statistical analysis of cell apoptosis distribution based on Figure 2A flow cytometry analysis. Date are presented as means ± SD (n = 3). ***$p < 0.001$ vs. LPS. $^{###}P < 0.001$ vs. AOzyme@ACM (one-way ANOVA with Bonferroni post hoc test).

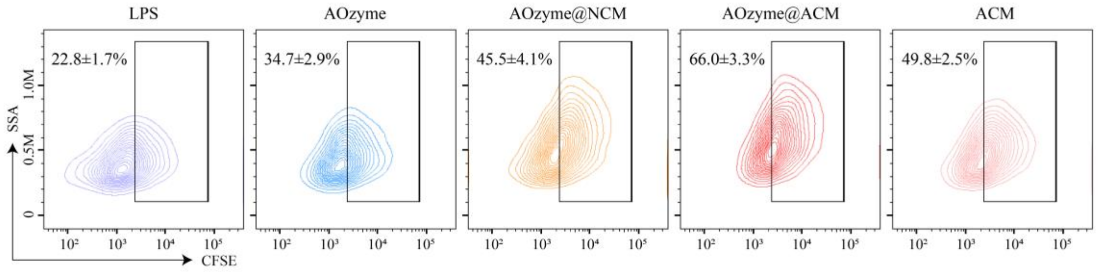

Figure S18. Flow cytometry analysis to assess efferocytosis of Raw264.7 cells *in vitro*. Apoptotic Jurkat cells labeled with CFSE. Date are presented as means ± SD (n = 5).

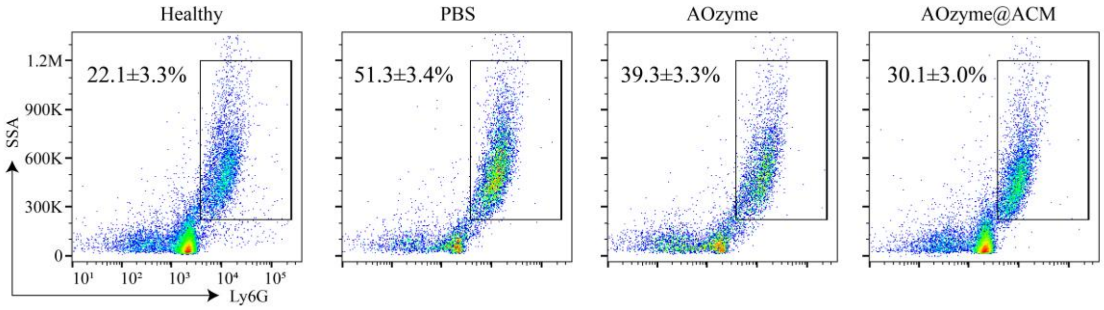

Figure S19. Neutrophils in BALF were identified by Ly6G staining and detected by flow cytometry. Date are presented as means ± SD (n = 5).

References

[1] S. Wu, P. Wang, J. Qin, Y. Pei, Y. J. A. F. M. Wang, *ACS. Appl. Mater. Inter.* **2021**, 31, 2102160.
[2] J. Zhao, S. Wu, J. Qin, D. Shi, Y. J. A. A. M. Wang, *ACS. Appl. Mater. Inter.* **2018**, 10, 41986.
[3] G. Yang, L. Xu, Y. Chao, J. Xu, X. Sun, Y. Wu, R. Peng, Z. Liu, *Nat. Commun.* **2017**, 8, 902.
[4] a)T. Liu, B. Xiao, F. Xiang, J. Tan, Z. Chen, X. Zhang, C. Wu, Z. Mao, G. Luo, X. Chen, J. Deng, *Nat. Commun.* **2020**, 11, 2788; b)J. Xiong, Y. Wang, Q. Xue, X. J. G. C. Wu, *Green. Chem.* **2011**, 13, 900.
[5] K. Xie, M. Varatnitskaya, A. Maghnouj, V. Bader, K. F. Winklhofer, S. Hahn, L. I. Leichert, *Redox. Biol.* **2020**, 28, 101344.
[6] Q. Zhang, D. Dehaini, Y. Zhang, J. Zhou, X. Chen, L. Zhang, R. H. Fang, W. Gao, L. Zhang, *Nat. Nanotechnol.* **2018**, 13, 1182.
[7] a) L. Lin, J. Song, L. Song, K. Ke, Y. Liu, Z. Zhou, Z. Shen, J. Li, Z. Yang, W. Tang, G. Niu, H. Yang. *Angew. Chem. Int. Edit*. **2017**, 130, 4996-5000; b) Z. Wang, H. Zhao, H. Qi, X. Liu, Y. Liu. *Environ. Technol*. **2019**, 40, 1138-1145.
[8] G. Matute-Bello, G. Downey, B. B. Moore, S. D. Groshong, M. A. Matthay, A. S. Slutsky, W. M. Kuebler, *Am. J. Resp. cell. Mol. biology* **2011**, 44, 725.
